# Experimental evolution is not just for model organisms

**DOI:** 10.1371/journal.pbio.3001587

**Published:** 2022-03-30

**Authors:** Anthony Burnetti, William C. Ratcliff

**Affiliations:** School of Biological Sciences, Georgia Institute of Technology, Atlanta, Georgia, United States of America

## Abstract

This Primer explores the implications of a new PLOS Biology study in which the authors evolve simple multicellularity in Sphaeroforma arctica, a unicellular relative of animals; this work establishes a new and open-ended avenue for examining the evolution of multicellularity at the base of the metazoa.

## Teaser

In a new paper published in *PLOS Biology*, Dudin and colleagues evolve simple multicellularity in *Sphaeroforma arctica*, a unicellular relative of animals. This work establishes a new and open-ended avenue for examining the evolution of multicellularity in an important but understudied group of organisms.

Our view of life on Earth is just a snapshot of an ancient and dynamic process. From observations spanning the scale of human lifetimes, we find ourselves trying to understand changes that have unfolded over hundreds of millions or even billions of years, a task akin to trying to piece together what happened in April from a half-second observation in October.

Our understanding of the patterns created by evolution has historically led our understanding of its processes. For instance, Carl Linnaeus inadvertently began systematically describing life’s evolutionary history over a hundred years before the publication of Charles Darwin’s *On the Origin of Species* [1], and it was another century before we began to understand the molecular details of evolutionary change. Resolving evolutionary dynamics can be a challenge even in extant populations, and our ability to infer the dynamics of ancient processes is intrinsically limited.

In recent decades, microbial experimental evolution has emerged as a particularly powerful tool for examining evolutionary dynamics. In these experiments, microbes are evolved under laboratory conditions and typically cryopreserved at regular intervals. Such experiments record unprecedented evolutionary detail; in principle, nearly every molecular change can be interrogated. The modern field of microbial experimental evolution can largely trace its origins to Richard Lenski’s long-term evolution experiment (LTEE). This experiment has been continuously running for 34 years (save 2 brief interruptions due to moving labs and then Coronavirus Disease 2019 [COVID-19]), and its *Escherichia coli* have undergone approximately 75,000 generations of growth. This project has yielded multiple novel insights into evolutionary dynamics (for instance, showing that fitness shows diminishing returns but appears unbounded [2] or revealing the precise molecular details of how novel traits arise [3]).

Experimental evolution has also been used to investigate a particular major evolutionary innovation: the origin of multicellularity. An ongoing long-term experiment in yeast is being used to examine how groups of cells form and adapt as multicellular individuals [4,5], and experimental evolution has been used to show how cooperative metabolism [6] and predation escape [7,8] can drive the evolution of simple multicellularity. These experiments have helped to shape the way we think about this transition, showing that a process once thought to be rare and highly constrained may in fact be relatively easy. However, like nearly all experimental evolution projects, these have relied on experimentally tractable model organisms rather than those with ecological or evolutionary connections to biosphere-altering major evolutionary transitions. Model organisms come with many distinct advantages for these experiments, not the least of which are standardized culture methods, simple genetic manipulation, and a profound wealth of prior knowledge about these organisms that has accumulated over decades. But they also come with inescapable limitations—laboratory evolution of yeast cannot directly answer questions about cell-to-cell adhesion without a fungal cell wall, for example, nor could experimental evolution of *E*. *coli* tell you something about the eukaryotic cell division cycle.

**Fig 1:**
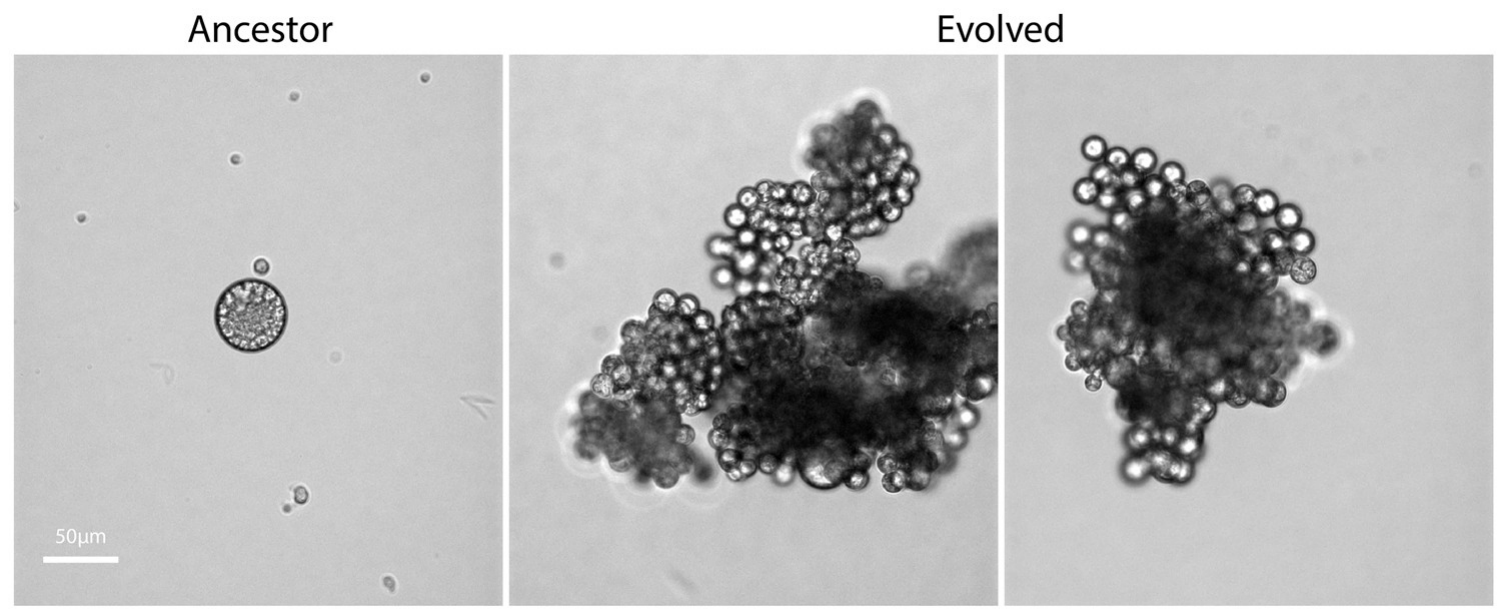
**Ancestral (left) and evolved (middle and right) *Sphaeroforma arctica*.** The ancestor on the left is shown in the largest phase of its life cycle. After several weeks of selection for rapid sedimentation, the isolates on the right evolved to form clonal multicellular groups. *Photo provided by Omaya Dudin*, *Swiss Institute for Experimental Cancer Research (ISREC)*.

In their latest article, “Regulation of sedimentation rate shapes the evolution of multicellularity in a close unicellular relative of animals” [9], Dudin and colleagues have taken experimental evolution into a new and exciting terrain. Moving out of the realm of model organisms, they examine the evolution of multicellularity in the icthyosporean *Sphaeroforma arctica*. This microbe hails from the Holozoa, a group of microbes closely related to animals (sharing a common ancestor approximately 1.1 billion years ago [10]). *S*. *arctica* spends most of its life cycle as a unicellular organism, although it has a transient syncytial and multicellular phase like many microbes. By applying selection for rapid sedimentation through liquid media, a process that efficiently selects on organism size and has previously been used to drive the evolution of multicellularity in *Saccharomyces cerevisiae* [4,5], they are able to evolve this primarily unicellular relative of animals to form large, clonal, consistently multicellular groups within a matter of weeks (Fig 1).

Via sequencing, they found that genes tied to the secretory pathway, signal transduction, cytokinesis, cell cycle control, and multiple transcription factors contributed to these phenotypes. Strikingly, they also found that, in addition to multicellular groups forming via incomplete cell wall separation (a common route to clonal multicellularity), selection for rapid sedimentation led to fundamental shifts in the timing of nuclear versus cytoplasmic division, concentrating nuclear material in the cells and increasing their buoyant density. This recapitulated natural diversity seen in related icthyosporean species, suggesting that buoyancy regulation may be an ecologically relevant trait in the Holozoa, not just a convenient means of selecting for multicellular groups in the lab.

The most important aspect of this work is how it moves beyond classic laboratory model organisms, allowing us to answer questions about clades that have had a particularly large impact on the course of life on Earth. Prior work evolving simple multicellularity in model organisms has played a key role in our understanding of how multicellularity arises in principle. But many of the questions one would like to answer about the evolution of multicellularity are lineage dependent, relying on the details of the unicellular ancestor’s cell biology and ecology. By working with a relative of animals, this paper opens a new chapter in experimental evolution.

The Holozoa are complex and diverse organisms, possessing extracellular matrix, cell–cell signaling, cell–cell adhesion molecules, and gene regulatory pathways once thought to be unique to animals [11,12]. Experimental evolution of multicellularity in a Holozoan (even if, like *S*. *arctica*, it is not an actual ancestor of animals) will allow novel questions to be explored. How “potentiated” are these unicellular organisms for the evolution of multicellularity, relative to model organisms like yeast or *Chlamydomonas*? How are unicellular life history traits co-opted for the evolution of increasingly complex multicellular behaviors, like cellular differentiation? What selective pressures might have driven the transition to multicellularity in the clade that includes animals and their closest relatives? Answering these questions will require not only patient and clever laboratory evolution experiments, but also the development of cell biological and genetic tools in these relatively understudied organisms, a step that will be necessary for if these experiments are to fully utilize the potential of long-term experimental evolution.

Studying major evolutionary transitions using only extant and fossilized organisms risks missing important information. These events represent complex innovations with many steps, the earliest of which are unlikely to have been preserved geologically as fossils or biologically as early branching “living fossils.” Many are also rare, with some even known as “evolutionary singularities” [13]—unique events that are difficult to study due to a lack of parallel transitions to compare against. Experimental evolution gives researchers like Dudin and colleagues the ability to create their own parallel transitions and to observe in detail the accumulation of small steps that together appear mighty in the imperfect mirror of the fossil record. We could not be more excited that new researchers are joining this community and bringing with them new organisms and questions.

## References

[1] Linnaeus C. Systema naturae. Vol. 1. Stockholm Laurentii Salvii. p. 1758.

[2] Lenski RE, Rose MR, Simpson SC, Tadler SC. Long-term experimental evolution in Escherichia coli. I. Adaptation and divergence during 2,000 generations. Am Nat. 1991;138(6):1315–41.

[3] Blount ZD, Barrick JE, Davidson CJ, Lenski RE. Genomic analysis of a key innovation in an experimental Escherichia coli population. Nature. 2012;489(7417):513–8. doi: 10.1038/nature11514 22992527 PMC3461117

[4] Ratcliff WC, Denison RF, Borrello M, Travisano M. Experimental evolution of multicellularity. Proc Natl Acad Sci U S A. 2012;109(5):1595–600. doi: 10.1073/pnas.1115323109 22307617 PMC3277146

[5] Bozdag GO, Zamani-Dahaj SA, Kahn PC, Day TC, Tong K, Balwani AH, et al. De novo evolution of macroscopic multicellularity. bioRxiv. 2021. 10.1038/s41586-023-06052-1 PMC10425966 37165189

[6] Koschwanez JH, Foster KR, Murray AW. Improved use of a public good selects for the evolution of undifferentiated multicellularity. Elife. 2013;2:e00367. doi: 10.7554/eLife.00367 23577233 PMC3614033

[7] Boraas ME, Seale DB, Boxhorn JE. Phagotrophy by a flagellate selects for colonial prey: a possible origin of multicellularity. Evol Ecol. 1998;12(2):153–64.

[8] Herron MD, Borin JM, Boswell JC, Walker J, Knox CA, Boyd M, et al. De novo origins of multicellularity in response to predation. Sci Rep. 2019;9(1):2328. doi: 10.1038/s41598-019-39558-8 30787483 PMC6382799

[9] Dudin O, Wielgoss S, New AM, Ruiz-Trillo I. Regulation of sedimentation rate shapes the evolution of multicellularity in a close unicellular relative of animals. PLoS Biol. 2022;20(3):e3001551. doi: 10.1371/journal.pbio.3001551 35349578 PMC8963540

[10] Parfrey LW, Lahr DJG, Knoll AH, Katz LA. Estimating the timing of early eukaryotic diversification with multigene molecular clocks. Proc Natl Acad Sci U S A. 2011;108(33):13624–9. doi: 10.1073/pnas.1110633108 21810989 PMC3158185

[11] Ros-Rocher N, Pérez-Posada A, Leger MM, Ruiz-Trillo I. The origin of animals: an ancestral reconstruction of the unicellular-to-multicellular transition. Open Biol. 2021;11(2):200359. doi: 10.1098/rsob.200359 33622103 PMC8061703

[12] Bråte J, Neumann RS, Fromm B, Haraldsen AAB, Tarver JE, Suga H, et al. Unicellular origin of the animal microRNA machinery. Curr Biol. 2018:28(20):3288–95.e5. doi: 10.1016/j.cub.2018.08.018 30318349 PMC6206976

[13] De Duve C. Singularities: landmarks on the pathways of life. Cambridge University Press; 2005.

